# On limits of contact tracing in epidemic control

**DOI:** 10.1371/journal.pone.0256180

**Published:** 2021-08-18

**Authors:** Tomasz Piasecki, Piotr B. Mucha, Magdalena Rosińska

**Affiliations:** 1 Institute of Applied Mathematics and Mechanics, University of Warsaw, Warszawa, Poland; 2 Department of Infectious Disease Epidemiology and Surveillance, National Institute of Public Health - National Institute of Hygiene, Warsaw, Poland; University of Oklahoma Norman Campus: The University of Oklahoma, UNITED STATES

## Abstract

Contact tracing and quarantine are well established non-pharmaceutical epidemic control tools. The paper aims to clarify the impact of these measures in evolution of epidemic. The proposed deterministic model defines a simple rule on the reproduction number R in terms of ratio of diagnosed cases and, quarantine and transmission parameters. The model is applied to the early stage of Covid19 crisis in Poland. We investigate 3 scenarios corresponding to different ratios of diagnosed cases. Our results show that, depending on the scenario, contact tracing prevented from 50% to over 90% of cases. The effects of quarantine are limited by fraction of undiagnosed cases. The key conclusion is that under realistic assumptions the epidemic can not be controlled without any social distancing measures.

## 1 Introduction

Given the lack of effective vaccine and treatment in 2020, the response to SARS-CoV-2 epidemic relied on traditional control measures, including a variety of travel restrictions and social distancing regulations [1]. While these measures helped to slow down the epidemic they came at significant economical and societal cost [2]. As an alternative an approach focusing on rapid diagnosis was recommended [3]. It was hoped that large-scale community testing coupled with contact tracing would allow to lift social distancing measures [4]. Of note, by isolating the asymptomatic contacts from their social networks, this strategy takes into account the pre-symptomatic and asymptomatic spread of the infection [5, 6], believed to be one of the key drivers of fast spread of the disease. As an example, wide spread testing in general population followed by isolation of the infected helped to reduce COVID-19 incidence by 90% during the first epidemic wave in an Italian village of Vo’Euganeo [7]. While a number of studies estimate the effects of different general social distancing measures on incidence, e.g. [1, 8–11], less is known about the impact of quarantine. Modelling confirms that effective testing is a necessary factor for this strategy to work [8, 12, 13], although studies differ in their conclusions to what extent this strategy would allow to relax the social restrictions. Hellewell et al. [14] investigated the potential of rapid isolation of cases and contact tracing to control the epidemic, finding that prohibitively high levels of timely contact tracing are necessary to achieve control. However, new technologies may offer sufficiently fast alternative to traditional contact tracing, in which case the epidemic could be still controlled by contact tracing [15]. In addition, a mixed strategy including a combination of contact tracing and social restrictions is usually applied.

This paper aims to define a deterministic SEIR-type population model describing the epidemic in classical terms of susceptible, exposed, infectious, removed and incorporating in addition the effects of quarantine. In order to validate our model in a setting in which measures to reduce contacts are in place, we apply it to investigate the role of quarantine during the first wave of COVID-19 epidemic in Poland. In our model the quarantine becomes a separate state that removes individuals from susceptible and exposed states. We show that the reproductive number in our model is given by a simple formula referring to the parameters of transmission and transition, but also to parameters describing the quarantine. We demonstrate that in a real life scenario (case study of Poland) the quarantine effectively reduces the growth of infectious compartment. Increasing the efficiency of contact tracing and testing may may to some extent compensate lifting up the social distancing restrictions.

## 2 Methods

### 2.1 The model

We introduce a modification of the classical SEIR model including effects of quarantine. Formally the model is described by a system of ordinary differential equations with delay dedicated to the quarantine.

The following states are included in the model:

*S*(*t*)—susceptible
*E*(*t*)—exposed (infected, not infectious)
*I*_*d*_(*t*)—infectious who will be diagnosed
*I*_*u*_(*t*)—infectious who will not be diagnosed
*R*_*d*_(*t*)—diagnosed and isolated
*R*_*u*_(*t*)—spontaneously recovered without being diagnosed
*Q*(*t*)—quarantined

The parameters include: *β*_*d*_ and *β*_*u*_—transmission rates for diagnosed and undiagnosed cases; *σ*—transition rate from the exposed state to infectious state; *κ*—diagnosis rate; *γ*_*d*_ and *γ*_*u*_—transition rate from infectious to non-infectious states (isolated or recovered) for diagnosed and undiagnosed cases; *θ*—proportion of infected among quarantined; *α*—the average number of quarantined contact of a single case; *T*—quarantine period.

**Fig 1:**
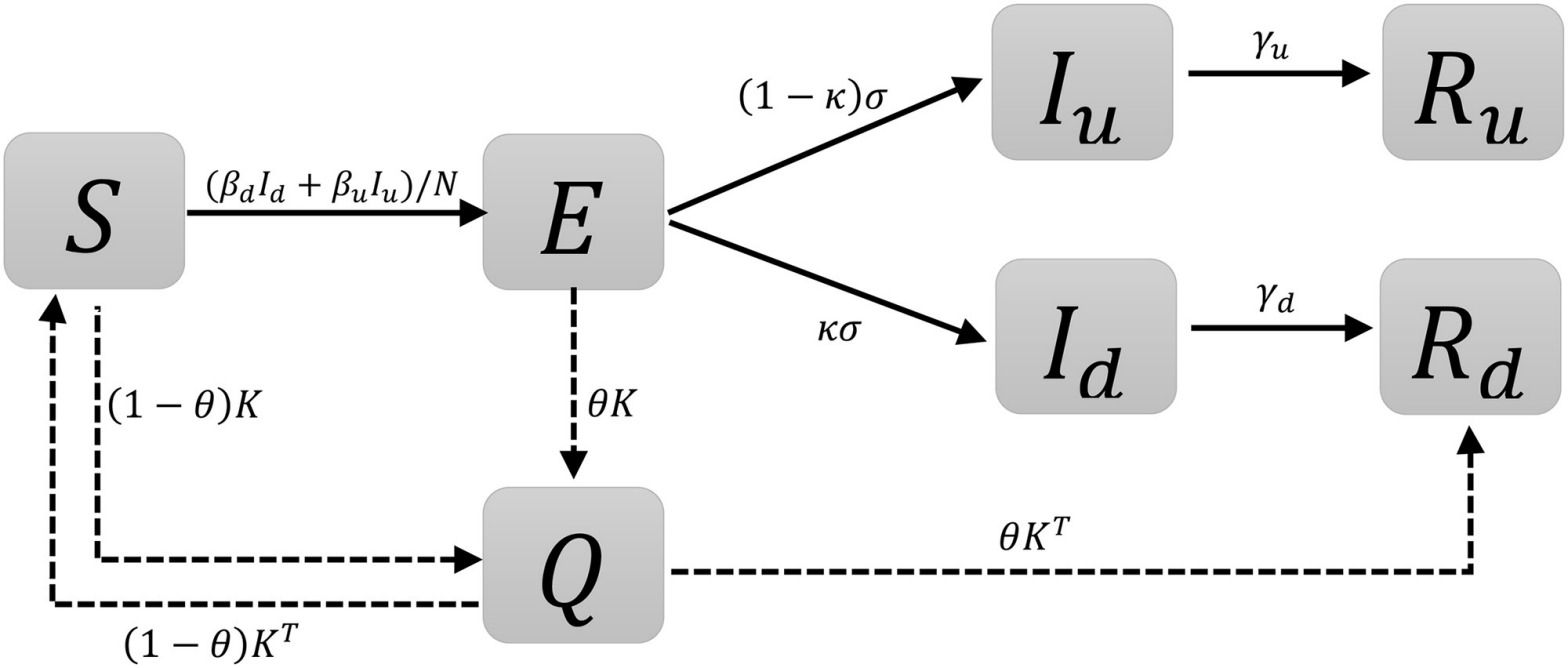
Schematic representation of the states included in the model. The solid lines represent the transition parameters and the dashed line indicate that the specific quantity is added.

The Fig 1 presents the schematic representation of the model. A susceptible individual (state *S*), when becoming infected first moves to the state *E*, to model the initial period, when the infected individual is not yet infectious. Next the cases progress to one of the infectious states *I*_*d*_ (infectious, who will be diagnosed) and *I*_*u*_ (infectious, who will never be diagnosed) at the rates *κσ* and (1 − *κ*)*σ*, respectively. Moving through the *I*_*d*_ pathway concerns these individuals who will be eventually detected by the health system, since they would meet the testing criteria, as relevant to the local testing policy, e.g. testing of people with noticeable symptoms. The quantity *I*_*u*_ shall be regarded as those who will not get detected by the health system, the undiagnosed infections, not necessarily asymptomatic or mild. We note that at the separation of the two *I* compartments, *I*_*d*_ and *I*_*u*_, is purely theoretical, based on their future development. Both *I*_*d*_ and *I*_*u*_ represent undiagnosed, infectious individuals. We make this distinction in order to be able to model separately the process of diagnosis and isolation that moves individuals from *I*_*d*_ to *R*_*d*_ and the process of spontaneous recovery that moves people from *I*_*u*_ to *R*_*u*_. With this interpretation the value of *κ* describes intensity of testing. In the classical setting, one thinks about *κ* as ratio between symptomatic and asymptomatic individuals. However, testing criteria may also include screening of selected asymptomatic groups, consequently *I*_*d*_ also includes asymptomatic infections found by the system supporting the interpretation of *κ* in terms of testing patterns.

The creation of state *E* is via *I*_*d*_ and *I*_*u*_ with transmission rates *β*_*d*_ and *β*_*u*_, respectively, normalized to the total population size *N* = *S* + *E* + *I*_*d*_ + *I*_*u*_ + *R*_*d*_ + *R*_*u*_ + *Q*, which is assumed to be constant in time, births and deaths are neglected.

The transition parameter *σ* is assumed identical for both groups, relating to the time between infection and becoming infectious. The infectious individuals from *I*_*d*_ then move to the state *R*_*d*_, which is the state of being diagnosed and isolated (and later recovered or deceased), with the rate *γ*_*d*_ corresponding to the observed time between onset and diagnosis. Hence we assume that an individual from *I*_*d*_ goes to *R*_*d*_ as he/she is detected by the system. On the other hand *R*_*u*_ contains people who spontaneously recovered with rate *γ*_*u*_. So *I*_*u*_ goes to *R*_*u*_ without notification of the system.

Let us now move to brief description of the main novelty of our model which is an additional state of being quarantined (*Q*). We consider the effective quarantine, which is the one applied before the individual becomes infectious. In the ideal situation, the quarantine is taken from the contacts of the diagnosed persons within 2–3 days of diagnosis of the index case, less then 4–5 days after the contact. In this idealized setting we take the quarantine from *E* and *S* only. Of course in real situation for some cases it can take longer, and the infected individuals are captured from *I*_*d*_ stata, but the number of such persons from *I*_*d*_ is small. We assume that such effect can be neglected. To mimic the situation of contact tracing, individuals can be put in quarantine (*Q*) from the state *S* (uninfected contacts) or the state *E* (infected contacts). These individuals stay in the quarantine for a predefined time period *T*. We assume that the number of people who will be quarantined depend on the number of individuals who are diagnosed. An average number of individuals quarantined per each diagnosed person is denoted as *α*.

However, as the epidemic progresses some of the contacts could be identified among people who were already infected, but were not previously diagnosed, i.e. the state *R*_*u*_. We note that moving individuals between the states *Q* and *R*_*u*_ has no effect on the epidemic dynamics (since the individuals in both compartments do not infect others), therefore only individuals from *S* and *E* are effectively quarantined. To take into account this situation, we reduce the average number of people put on quarantine by the factor $\frac{S(t)}{S\left(t\right)+R_{u}\left(t\right)}$.

Further, to acknowledge the capacity limits of the public health system to perform the contact tracing, we introduce a quantity *K*_*max*_, describing the maximum number of people who can be put in quarantine during one time unit.

We also assume that among the quarantined a proportion *θ* is infected. The quarantine process is determined by tracking of contacts of diagnosed people. Thus, by definition this tracked group has high risk of infection, higher than in general population. The factor *θ* gives a rate of infection among contacts of infected individuals, which we assume to be a stable quantity, depending on natural transmissibility of the virus. From that viewpoint the relative magnitude of *S* and *E* does not play an essential role in describing *θ*.

After the quarantine, the infected part *θK*(*t* − *T*) goes to *R*_*d*_ and the rest (1 − *θ*)*K*(*t* − *T*) returns to *S*. *K*(*t*) is defined by the formula in (1). The number of newly quarantined depend on the number of newly diagnosed. This is *γI*_*d*_(*t*) + *θK*(*t* − *T*). The last part, *θK*(*t* − *T*), represents the new diagnoses from the quarantine, so it does not contribute to defining *K*(*t*). *K*(*t*) is determined by newly diagnosed, not being on quarantine before diagnosis, i.e. *γI*_*d*_(*t*).

Taking all of the above into account, the model is described with the following SEIRQ system:

$$\begin{array}{c} \begin{array}{c} \dot{S}\left(t\right)=-\frac{S(t)}{N}\left(\beta_{d}I_{d}\left(t\right)+\beta_{u}I_{u}\left(t\right)\right)-\left(1-\theta \right)K\left(t\right)+\left(1-\theta \right)K\left(t-T\right),\\ \dot{E}\left(t\right)=\frac{S(t)}{N}\left(\beta_{d}I_{d}\left(t\right)+\beta_{u}I_{u}\left(t\right)\right)-\sigma E\left(t\right)-\theta K\left(t\right)\\ {\dot{I}}_{d}\left(t\right)=\kappa \sigma E\left(t\right)-\gamma_{d}I_{d}\left(t\right),\\ {\dot{I}}_{u}\left(t\right)=\left(1-\kappa \right)\sigma E\left(t\right)-\gamma_{u}I_{u}\left(t\right),\\ {\dot{R}}_{d}\left(t\right)=\gamma_{d}I_{d}\left(t\right)+\theta K\left(t-T\right),\\ {\dot{R}}_{u}\left(t\right)=\gamma_{u}I_{u}\left(t\right),\\ \dot{Q}\left(t\right)=K\left(t\right)-K\left(t-T\right),\\ \text{where}\;\;\;K\left(t\right)=\text{min}\left\{\frac{S(t)}{S\left(t\right)+R_{u}\left(t\right)}\alpha \gamma_{d}I_{d}\left(t\right),K_{max}\right\},\;\;\text{and}\;\;\;\alpha ,\beta_{d},\beta_{u},\gamma_{d},\gamma_{u},\theta ,T\geq 0. \end{array} \end{array}$$ (1)

We assume that the parameters *α*, *β*_*d*_, *β*_*u*_, *θ*, *γ*_*u*_ and *γ*_*d*_ depend on the country and time-specific public health interventions and may therefore change in time periods. Due to proper interpretation of the equation on *E* we require that *β*_*d*_ ≥ *θαγ*_*d*_ to ensure positiveness of *E*. Initial data for the system are discussed in S1 Appendix.

### 2.2 Basic reproductive number, critical transmission parameter β*

Based on the general theory of SEIR type models [16], we introduce the reproductive number

$$\begin{array}{c} R=\kappa (\frac{\beta_{d}}{\gamma_{d}}-\theta \alpha )+\left(1-\kappa \right)\frac{\beta_{u}}{\gamma_{u}}. \end{array}$$ (2)

It determines the stability of the system as R<1 and instability for R>1 (the growth/decrease of epidemic). This quantity explains the importance of testing (in terms of *κ*) and quarantine (in terms of *α*), but also gives an indication on levels of optimal testing and contact tracing. We underline that this formula works for the case when the capacity of the contact tracing has not been exceeded (*K*(*t*) < *K*_*max*_). We shall emphasize the formal mathematical derivation holds for the case when *I* and *E* are small comparing to *S*, see the S1 Appendix. Therefore the complete dynamics of the nonlinear system (1) is not fully determined by (2).

The critical value R=1 defines the level of transmission which is admissible, taking into account the existing quarantine policy, in order to control epidemic. As the level of transmission depends on the level of contacts, this provides information on the necessary level of social distancing measures. The formula (2) indicates that improving the contact tracing may compensate relaxation of contact restrictions. The key quantity is *θα*. Indeed the system with the quarantine has the same stability properties as one without *K*, but with the new transmission rate $\beta_{d}^{new}=\beta_{d}-\theta \alpha \gamma_{d}$. In order to guarantee the positiveness of *E*, $\beta_{d}^{new}$ must be nonnegative. It generates the constraint

$$\begin{array}{c} \theta \alpha \gamma_{d}\leq \beta_{d}. \end{array}$$ (3)

The above condition also implies the theoretical maximal admissible level of quarantine. We define it by improving the targeting of the quarantine, i.e. by the highest possible level of *θ*:

$$\begin{array}{c} \theta_{max}=\frac{\beta_{d}}{\gamma_{d}\alpha }. \end{array}$$ (4)

The effect of the increase in *θ* or in *α* play the same role at the level of linearization (small *I*, *E*). In general it is not the case and for the purpose of our analysis we fix *α*.

For our analysis we assume *β*_*d*_ = *β*_*u*_ = *β*. The reason is that, both *I*_*d*_ and *I*_*u*_ contain a mixture of asymptomatic and symptomatic cases and although there might be a difference we lack information to quantify this difference. Then using formula (2) we compute critical values β*(*κ*, *θ*, *α*) defined as

$$\begin{array}{c} R\left(\beta^{*}\right)=1,\;\;\text{namely}\;\;\beta^{*}\left(\kappa ,\theta ,\alpha ,\gamma_{d},\gamma_{u}\right)=\frac{\left(1+\theta \alpha \kappa \right)\gamma_{d}\gamma_{u}}{\gamma_{u}\kappa +\gamma_{d}\left(1-\kappa \right)}. \end{array}$$ (5)

It shows the upper bound on transmission rate *β* which still guarantees the suppression of pandemic. We shall omit the dependence on *γ*_*d*_, *γ*_*u*_ as these are fixed in our case, and denote briefly β*(*κ*, *θ*, *α*).

In the case of maximal admissible quarantine (4) we obtain

$$\begin{array}{c} \beta^{*}\left(\theta_{max},\kappa \right)=\frac{\gamma_{u}}{1-\kappa }, \end{array}$$ (6)

which can be regarded as theoretical upper bound for *β* if we assume “optimal admissible” quarantine for fixed *κ*, for which the epidemic could be still controlled. It must be kept in mind though that the condition (3) means that we are able to efficiently isolate all persons infected by every diagnosed, therefore is unrealistic. The resulting β*(*θ*_*max*_, *κ*) should be therefore considered as a theoretical limit for transmission rate.

### 2.3 Fitting procedure

All simulations are performed using GNU Octave (https://www.gnu.org/software/octave/). The underlying tool for all computations is a direct finite difference solver with a 1 day time step.

#### 2.3.1 Basic assumptions for data fitting

We estimate the transmission rates *β* by fitting the model predictions to the data on the cumulative number of confirmed cases. Since people with confirmed diagnosis are efficiently isolated, they are immediately included into *R*_*d*_. Therefore, the quantity fitted to the data is *R*_*d*_(*t*).

The crucial assumption behind our approach is that the parameter *β* changes twice during the period of analysis. The reason is that we can distinguish two important time points in the development of epidemic in Poland. The first case of COVID-19 in Poland was diagnosed on March 3rd. Social distancing measures were rapidly introduced during the week of 9—13th March including closure of schools and universities, cancellation of mass events and closure of recreation facilities such as bars, restaurants, gyms etc. as well as shopping malls. Religious gatherings were limited. Finally, borders were closed for non-citizens [17]. These measures were fully in place on March 16th. As we do not take migration into account in our model, we assume that the effect of border closing is reflected in *β*. Further, beginning at March 25th restrictions on movement and travel were introduced (lock-down). Wearing face covers became obligatory on April 14th. The restrictions were gradually lifted beginning at April 20th.

For simplicity we comprise the effect of above measures in two jump changes in *β* in *t* ∈ {*t*_1_, *t*_2_} and choose *t*_1_ = 14, *t*_2_ = 28. With *t* = 1 corresponding to March 3 it means small delay with respect to the above dates which can be justified by the fact that new cases are reported with a delay of approximately 2 days.

#### 2.3.2 Choice of fixed parameters (Table 1)

**Table 1:** Fixed parameters used in the model.

Parameter	Value	Source
*σ*	$\frac{1}{3.5}$	Literature: incubation time [18–20] + presymptomatic spread [5, 6, 21]
*γ*_*d*_	$\frac{1}{5.5}$	Observed data: appendix
*γ*_*u*_	$\frac{1}{10}$	Literature: [22], WHO mission report from China
*κ*	{0.2; 0.5; 0.8}	Literature: proportion asymptomatic or undocumented [7, 23–25]
*θ*	0.006	Observed data: appendix
*α*	75	Observed data: appendix
*K*_*max*_	50 000	2 × the maximum level observed so far (arbitrary decision)

The parameters *σ*, *γ*_*u*_ represent the natural course of infection and their values could be based on the existing literature. The parameter *σ* describes the rate of transition from non-infectious incubation state *E* into the infectious states *I*_*d*_ or *I*_*u*_. The value of *σ* takes into account the incubation period and presymptomatic infectivity period. *γ*_*u*_ relates to the period of infectivity, which we select based on the research regarding milder cases, assuming that serious cases are likely diagnosed. Further, *κ* is a parameter related both to the proportion of asymptomatic infection and the local testing strategies. Since the literature findings provide different possible figures, for *κ* we examine three different scenarios.

Parameters *γ*_*d*_, *θ* and *α* are fixed in our model for the purpose of data fitting, but informed by available data. One of the scenarios of future dynamics of the epidemic (section 3.3) considers possible increase of *θ*. Parameter *γ*_*d*_ was estimated basing on time from onset to diagnosis for diagnosed cases, and *θ* as rate of diagnosed among quarantined. Furthermore we fix the parameter *α* by comparing the number of quarantined people obtained in simulations with actual data. The capacity level of public health services is set in terms of possible number of quarantined per day *K*_*max*_, as double the level observed so far. Detailed justification of the values of fixed parameters collected in the following table, is given in the S1 Appendix.

#### 2.3.3 Optimization algorithm

In order to fit the values *β*_1_, *β*_2_, *β*_3_ we use a standard gradient descent algorithm. The error function is defined as mean square difference between the cumulative number of diagnoses and the *R*_*d*_(*t*) predicted from the model.

For the initial values the error function is optimized only for a limited number of possible conditions, as these mostly impact *β*_1_, which is less relevant for future predictions. To estimate confidence intervals we use a method of parametric bootstrap. The optimisation procedures are described in the S1 Appendix, where we also show precise errors of data fitting.

#### 2.3.4 Dataset

The data series contains cumulative number of confirmed cases of COVID-19 in Poland from March 3 (first confirmed case in Poland) till April 26, which amounts to 54 observations. The data are taken from official communications of the Ministry of Health. As explained in Table 1 and the S1 Appendix additional data sources were used for choosing *θ*, *α* and *γ*_*d*_.

## 3 Results

### 3.1 Estimation of parameters and “no-change” scenario predictions

**Fig 2:**
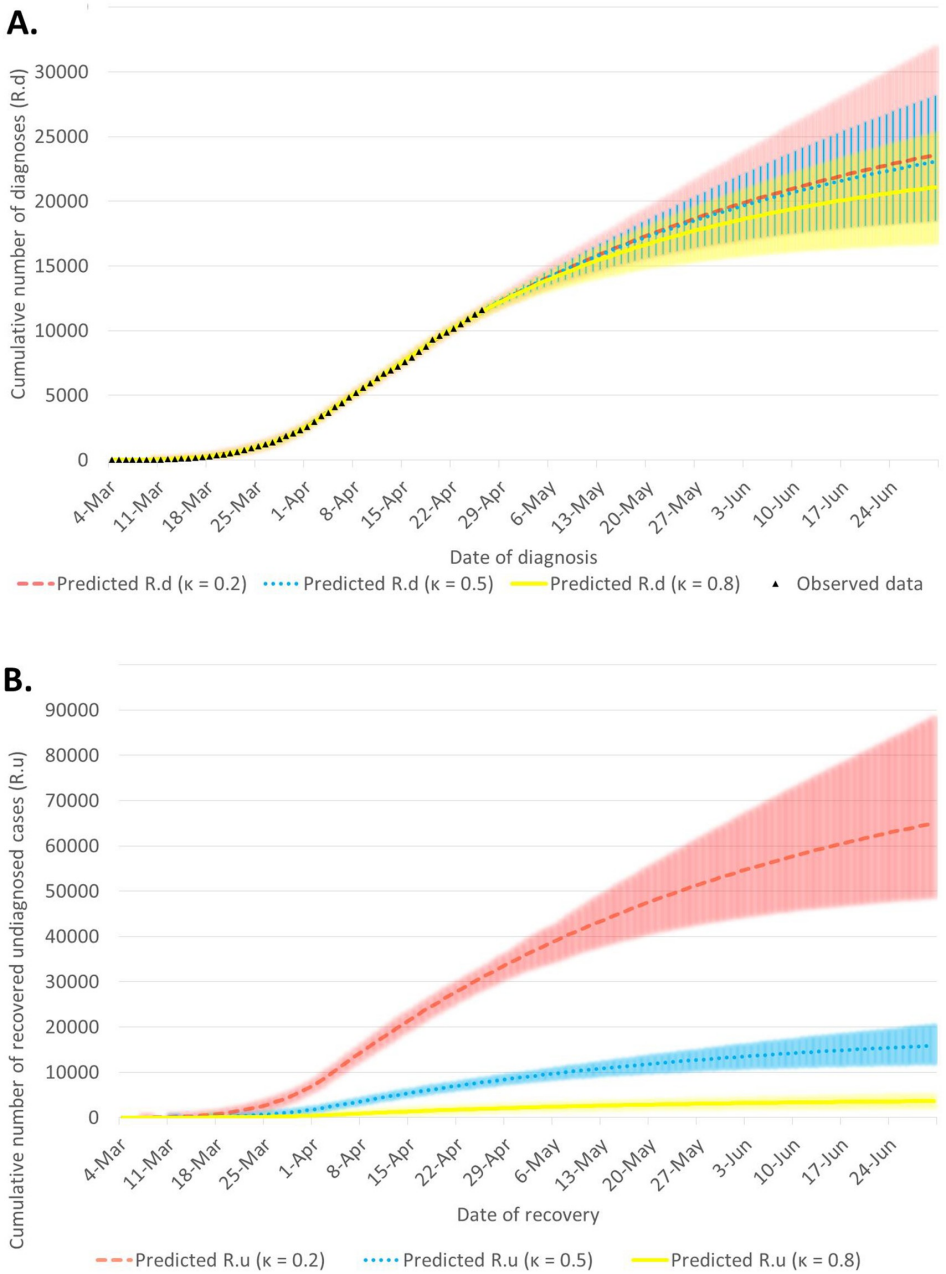
Results of model fit to cumulative diagnosed cases (*R*_*d*_) for *κ* = 0.2, 0.5, 0.8 (panel A) and corresponding predictions for undiagnosed, recovered compartment, *R*_*u*_ (panel B). Coloured shades correspond to 95% confidence intervals for the respective colour line.

In Table 2 we show estimated values of *β*_*i*_, where *i* = 1, 2, 3 correspond to the time intervals when different measures were in place, and the R for the third time interval. Given the social distancing measures in place early April 2020, as well as the quarantine levels, the reproductive number was below 1, independently of the value of *κ*, which relates to testing effectiveness. The Fig 2 shows the fit of the models assuming different levels of *κ*. Good fit is found for all three models although predictions start to differ in the middle-term prognosis.

**Table 2:** Estimated values of *β*_*i*_ and values of R corresponding to the latest estimation period with 95% confidence intervals.

	*κ* = 0.2	*κ* = 0.5	*κ* = 0.8
*β*_1_	0.635	0.684	0.738
	(0.569, 0.701)	(0.611, 0.744)	(0.672, 0.812)
*β*_2_	0.332	0.383	0.442
	(0.288, 0.397)	(0.336, 0.443)	(0.4, 0.514)
*β*_3_	0.099	0.132	0.175
	(0.081, 0.118)	(0.11, 0.149)	(0.147, 0.214)
$R(\beta_{3},0.006,75)$	0.817	0.802	0.772
	(0.651, 0.977)	(0.648, 0.915)	(0.569, 0.874)

**Fig 3:**
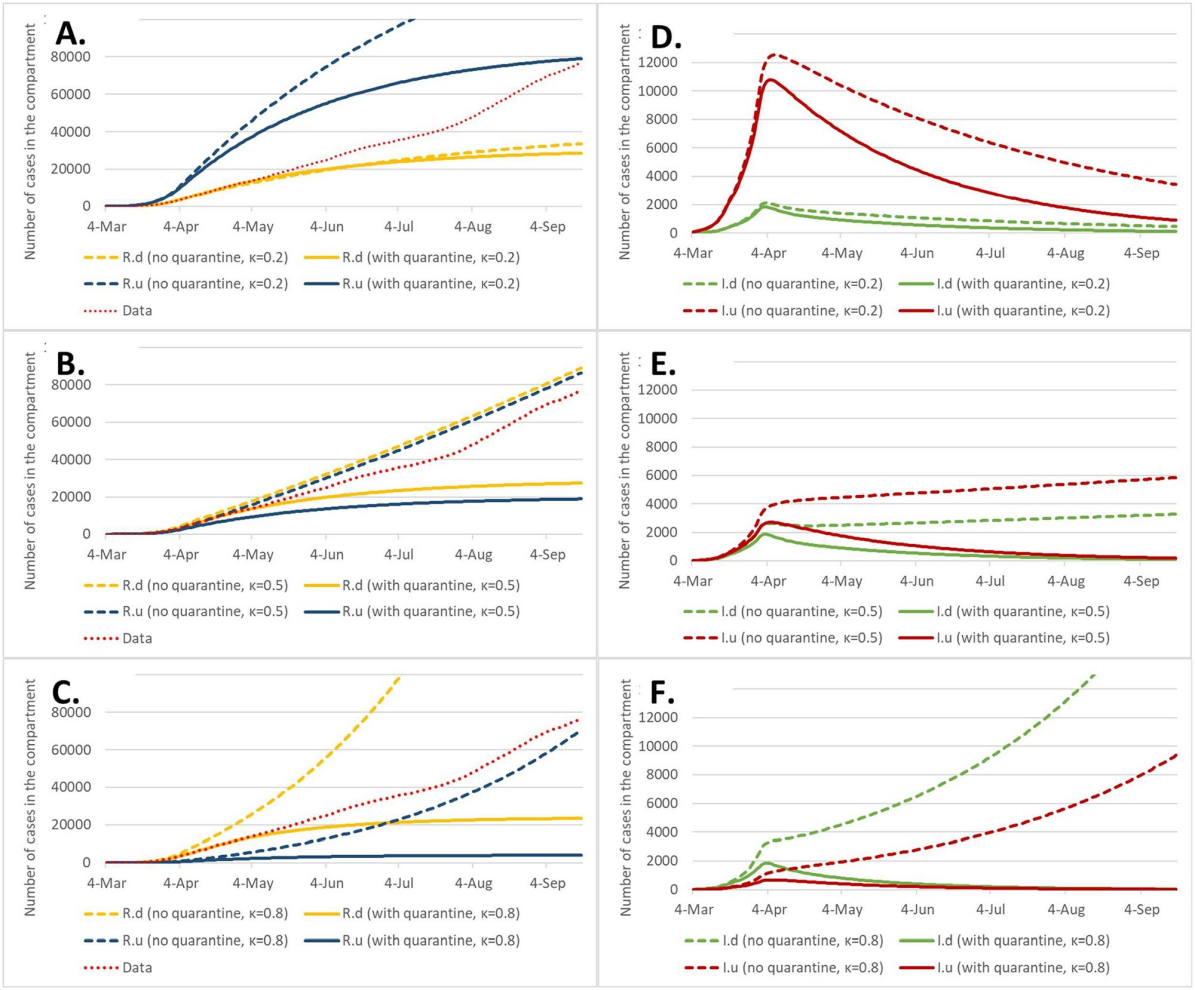
Predicted values of *R*_*d*_, *R*_*u*_ (panels A—C) and *I*_*d*_, *I*_*u*_ (panels D—E), as depending on the value of *κ* and whether or not the quarantine is implemented. For *t* > 54 *β* = *β*_3_ estimated for each *κ*, with the same quarantine parameters or without quarantine at all.

We proceed with predictions assuming that the restrictions are continued, i.e. keeping *β* = *β*_3_ (note that the estimated *β*_3_ is different for each *κ*). We calculate the epidemic duration (*t*_*max*_), the peak number of infected ($I_{d}^{max},I_{u}^{max}$) and the final size of the epidemic (*R*_*d*_(*t*_*max*_), *R*_*u*_(*t*_*max*_)). In order to show the influence of quarantine we compare the situation with quarantine, keeping the same *θ*, *α*, to the situation without quarantine, setting *αθ* = 0. The results of the development of the epidemic during the first 120 days are shown on Fig 3. For reference this figure also includes data observed at latter time.

For *κ* = 0.2 the difference between the scenarios with and without quarantine is visible but not striking. However for *κ* = 0.5 and *κ* = 0.8 a bifurcation in the number of new cases occurs around *t* = 40 leading to huge difference in the total time of epidemic and total number of cases. These values are summarized in the Table 3. We note that given the epidemic state in the first half of April 2020 for all values of *κ* the model predicts epidemic extinction both with quarantine and without quarantine. However, since the epidemic is very near to the endemic state, the predicted duration is very long, especially if no quarantine is applied. The data observed during the summer 2020 (dots “data” in Fig 3) exceed projections for the scenarios with quarantine for all values of *κ*, suggesting that the contact tracing efforts were reduced. The observed curve is the nearest to the scenario B of the Fig 3 (i.e. *κ* = 0.5), without quarantine. This corresponds to release of restrictions and weakened of contact tracing in that time.

**Table 3:** Duration of epidemic (*t*_*max*_) in days, the final values of *R*_*d*_ and *R*_*u*_, in thousands (*R*_*d*_(*t*_*max*_), *R*_*u*_(*t*_*max*_)) and peak values of *I*_*d*_ and *I*_*u*_, in thousands ($I_{d}^{max},I_{u}^{max}$) according to quarantine and testing scenarios.

*κ*	quarantine factors	*R*_*d*_(*t*_*max*_)	*R*_*u*_(*t*_*max*_)	$I_{d}^{max}$	$I_{u}^{max}$	*t*_*max*_
0.2	*θ* = 0.006, *α* = 75	31	85	1.9	10.8	450
	*θ*, *α* = 0	44	175	2.1	12.5	830
0.5	*θ* = 0.006, *α* = 75	29	20	1.9	2.7	330
	*θ*, *α* = 0	1078	1078	5.1	9.2	3200
0.8	*θ* = 0.006, *α* = 75	24	4	1.9	0.7	230
	*θ*, *α* = 0	6317	1579	10.6	47.6	1280

### 3.2 Critical β* for different case detection levels

Using the formula (5) we compute critical values β*. In Table 4 we show the values of β*(*κ*, 0.006, 75) and for convenience recall also estimated values of *β*_3_ and R, listed already in Table 2. Moreover we compute β*(*κ*, 0, 0), i.e. without quarantine and show values of R for our estimated values of *β*_3_ and the same *γ*_*d*_, *γ*_*u*_ but without quarantine. Comparing the estimated values of *β*_3_ (Table 4) for all cases of *κ* are only slightly below β*.

**Table 4:** Values of β* and $R(\beta_{3})$ with quarantine (*i* = 0.006, *α* = 75) and without quarantine.

*κ*	*β*_3_	β*(*κ*, 0.006, 75)	$R(\beta_{3},\kappa ,0.006,75)$	β*(*κ*, 0, 0)	$R(\beta_{3},\kappa ,0,0)$	*β*_3_ − *θαγ*_*d*_
0.2	0.099	0.12	0.817	0.11	0.907	0.018
0.5	0.132	0.158	0.802	0.129	1.03	0.051
0.8	0.175	0.211	0.772	0.155	1.132	0.074

Eliminating the quarantine, for the estimated values of *β*_3_, we have different situations depending on the actual value of *κ*. In case *κ* = 0.2, so assuming that currently only 20% of infections are diagnosed, the low values of R are due to low *β*_3_ rather than the effect of quarantine (controlling epidemic by social contact restrictions). In effect even if we remove the quarantine we have still R<1, but very close to 1. On the other hand if *κ* = 0.5 or *κ* = 0.8 we estimate higher *β*_3_, which corresponds to the situation of controlling the epidemic by extensive testing and quarantine. For these cases, if we remove the quarantine, we end up with R>1. The quantity *β*_3_ − *θαγ*_*d*_ represents effective transmission rate due to diagnosed cases. In particular it shows by how much the transmission could be reduced by improved contact tracing (*θα*) and faster diagnosis (*γ*_*d*_).

These results confirm that the higher is the ratio of undiagnosed infections, the weaker is influence of quarantine. In the next section we verify these results numerically.

### 3.3 Impact of quarantine at relaxation of social distancing

Our second goal is to simulate loosening of restrictions. In particular we want to verify numerically the critical thresholds β* listed in Table 4. For this purpose we assume that at *t* = 60 we change *β*. For each value of *κ* we consider 3 scenarios:

(a) Current level quarantine: i.e. quarantine parameters *θ* = 0.006, *α* = 75 are maintained;
(b) No quarantine is applied starting from *t* = 60;
(c) The maximal admissible quarantine is applied, meaning that $\theta_{max}=\frac{\beta }{\alpha \gamma_{d}}$ (see (2)). In this case *α* = 75. As long as the limit *K*_*max*_ is not reached there is no difference whether we increase *α* or *θ*, the decisive parameter is *αθ*. Increasing *α* would lead to reaching *K* = *K*_*max*_ earlier and hence worse outcomes.

**Fig 4:**
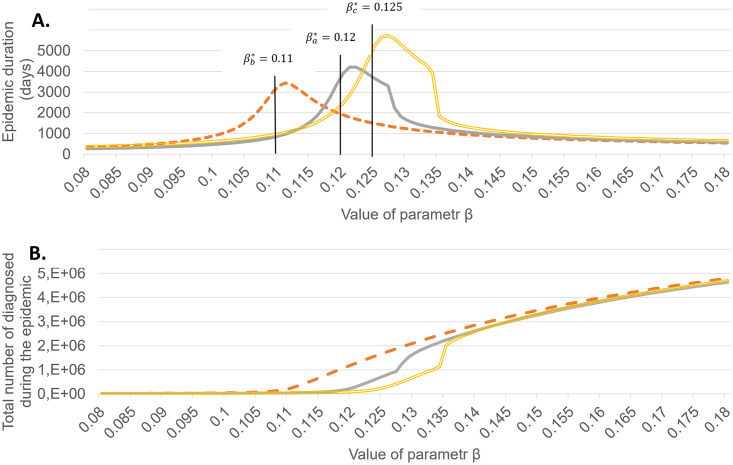
Duration of epidemic and the final epidemic size as dependent on *β*, for *κ* = 0.2. *β*^*a*^, *β*^*b*^, *β*^*c*^ correspond, respectively, to scenario (a), (b) and (c) outlined in the text.

**Fig 5:**
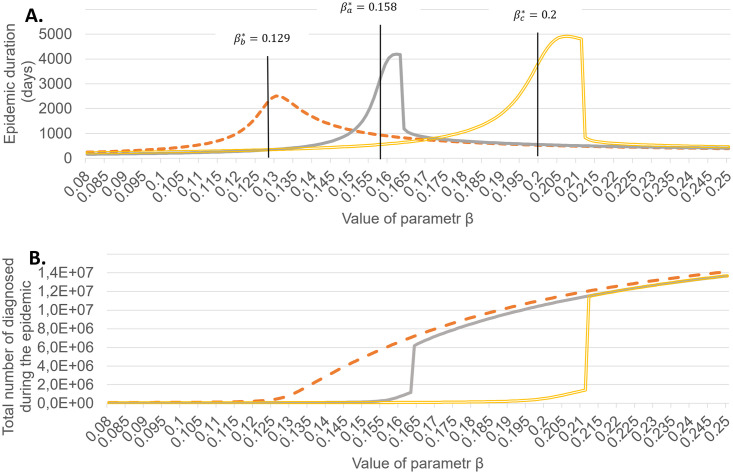
Duration of epidemic and the final epidemic size as dependent on *β*, for *κ* = 0.5. *β*^*a*^, *β*^*b*^, *β*^*c*^ correspond, respectively, to scenario (a), (b) and (c) outlined in the text.

**Fig 6:**
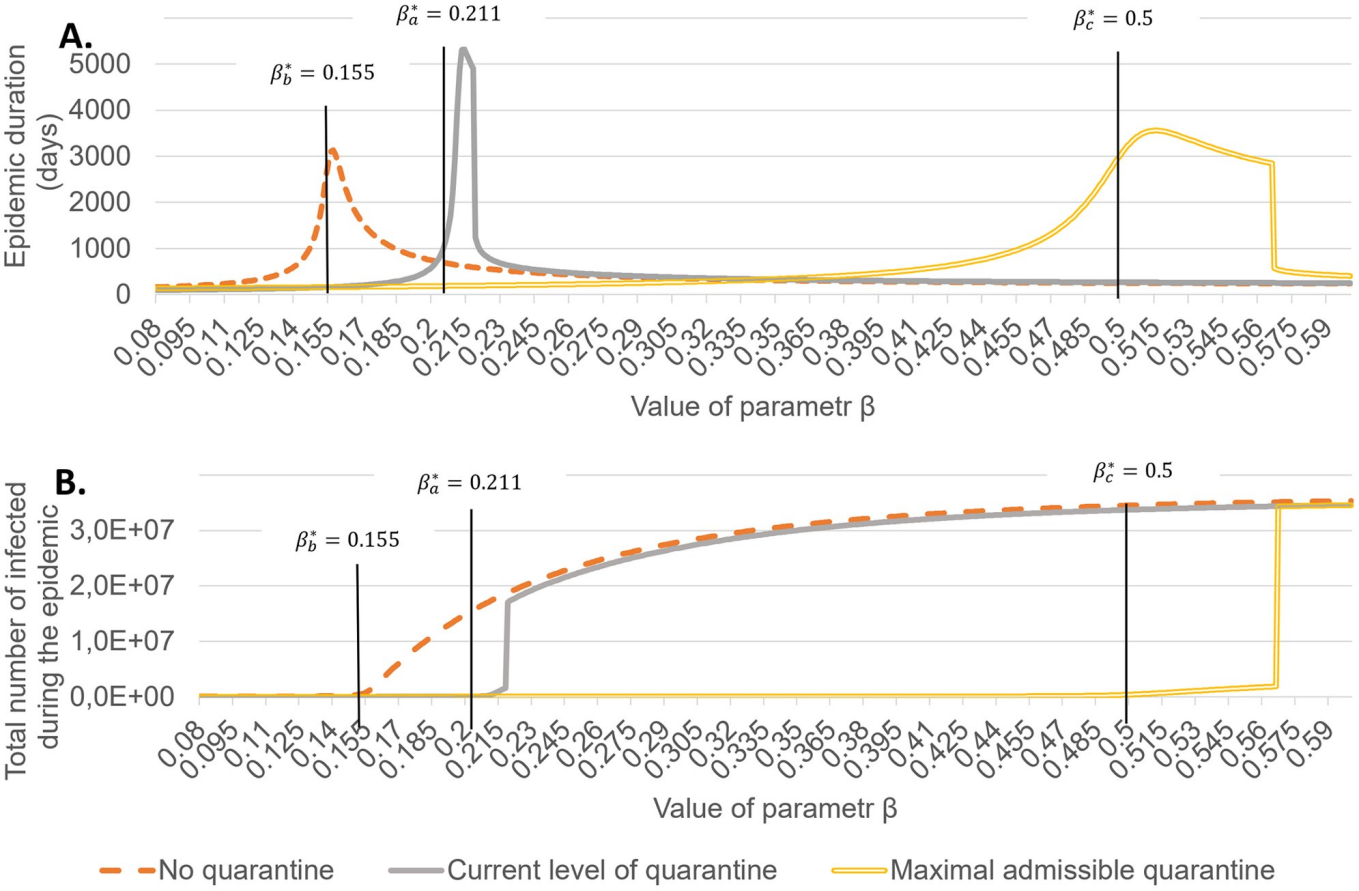
Duration of epidemic and the final epidemic size as dependent on *β*, for *κ* = 0.8. *β*^*a*^, *β*^*b*^, *β*^*c*^ correspond, respectively, to scenario (a), (b) and (c) outlined in the text.

Figs 4–6 show the final values of *R* = *R*_*d*_ + *R*_*u*_ and time till the end of epidemic depending on the value of *β* for *t* ≥ 60 for above 3 scenarios and different values of *κ*. The theoretical values of β* are shown by black lines.

The results confirm that around β* a rapid increase in the total number of infected occurs, coinciding with the peak total epidemic duration. Thus the numerical computations confirm that the critical β* calculated for the linear approximation in the section 2.2 are adequate, with a small bias towards lower values.

The case *κ* = 0.2 shows that the influence of quarantine is not high, even for the maximal admissible case, when we are able to efficiently isolate all persons infected by every diagnosed.

A striking feature in the behaviour of total number of infected are jumps for certain critical value of *β* observed for *κ* = 0.5 and *κ* = 0.8, both in case *θ* = 0.006 and *θ* = *θ*_*max*_. The values of *R*_*d*_ and *R*_*u*_ before and after these qualitative changes are summarized in Table 5.

**Table 5:** Critical values of *β* obtained in simulations and corresponding final numbers of diagnosed/undiagnosed (in thousands) and total time of epidemic.

	*β*	*R*_*d*_(*t*_*max*_)	*R*_*u*_(*t*_*max*_)	*t*_*max*_
*κ* = 0.5, *θ* = 0.006,	0.163	1171	811	4170
	0.164	6160	5875	1200
*κ* = 0.5, *θ* = *θ*_*max*_(*β*),	0.211	1423	666	4800
	0.212	11458	10971	840
*κ* = 0.8, *θ* = 0.006	0.218	1137	236	4740
	0.219	13706	3365	1060
*κ* = 0.8, *θ* = *θ*_*max*_(*β*)	0.566	1762	108	2850
	0.567	27602	6729	570

A closer investigation for these values of *β* shows that in all 4 cases the jump occurs for the first value of *β* for which the limit number of quarantined, *K*_*max*_ = 50000, is achieved. Notice that immediately after passing the threshold the values become very close to those without quarantine. Therefore the effect of quarantine is immediately and almost completely cancelled after passing the critical value of *β*. The transition is milder in the case *κ* = 0.2 which can be explained by the fact that the transition takes place for lower values of *β*.

Results of our simulations confirm the theoretical prediction that strengthening of quarantine allows to remain in a stable regime while increasing *β*. However, the margin in relaxation of restrictions is very narrow if we want to avoid a blow up of the number infections.

## 4 Discussion

We estimate the effects of contact tracing and quarantine during the initial period of COVID-19 epidemic in Poland. We show that these effects strongly depend on the efficiency of the testing systems and if only a small fraction of cases (e.g 20%) are detected, the effects of contact tracing effort are modest. Moreover, we show that in Poland it is not possible to return to the levels of social activity similar to those before the epidemic while controlling the epidemic solely by contact tracing strategy. In addition, lifting social restrictions would rapidly lead to exceeding the capacity of contact tracing services and when this happens the control is lost. It is therefore quite crucial to implement the aggressive contact tracing system, when the epidemic is still at low levels and it is possible to bring the epidemic to suppression phase. Our model offers a clear interpretation of the quarantine effect. The transmission rate due to diagnosed cases, *β*_*d*_, is decreased by the factor *θαγ*_*d*_ indicating that both the number of quarantined per diagnosed individual (*α*) and proper targeting of the quarantine (the infection rate among the quarantined *θ*) equally contribute to this factor. This quantifies the potential of a wide range of interventions to improve testing and contact tracing, as outlined in e.g. in ECDC recommendations [26]. In particular, as the number of people put in quarantine per each case and the infection rate among the quarantined impact R in similar fashion, our results support the recommendations to focus on the high risk contacts when the resources do not allow to follow all contacts.

Our model takes into consideration only the effective contact tracing, i.e. the situation when the infected contacts are identified and put in quarantine before they become infectious. People who are identified later would be modelled as passing through one of the *I* states to the *R* states. This means that the number of quarantined in our model can be also increased by faster contact tracing. The timely identification of contacts may be a significant challenge in the quarantine approach given that the incubation time can be as short as 2 days in 25% of cases [18]. The delays in manual contact tracing are usually at least 3 days [15] but this could be improved with digital contact tracing. Notably, mixed contact tracing strategies implemented in South Korea [27], Taiwan [28] or Singapore [29] indeed helped to control the epidemic without major disruptions of social activities.

We note that the quarantine effect relates only to transmission due to diagnosed cases. As expected, in order to control the epidemic the transmission due to undiagnosed cases has to be negligible. This can be controlled by general measures such as *lockdown*, which universally decrease the frequency of social contacts and are therefore likely to reduce *β*_*u*_. In our model the part of R representing transmission due to undiagnosed cases is scaled by (1 − *κ*), the parameter relating to the efficiency of the testing system. Again, the examples of Singapore as well as the Italian village of Vo’Euganeo show that the widespread testing complementing the efficient contact tracing was essential to suppress epidemic. Testing unrelated to epidemiological links decreases (1 − *κ*) factor, thus making the factors impacting transmission due to diagnosed cases, such as quarantine, more powerful to decrease R.

In line with this observation, the quarantine is estimated to be the most effective for the scenario in which most of the cases are diagnosed (*κ* = 0.8). Testing strategies that comprise testing of all individuals with symptoms of respiratory illness could theoretically identify up to 82% of infected, assuming they would all present to medical care. This could be coupled with random screening of high risk individuals, in e.g. health care workers, or—in case of high incidence—even random screening of entire community to achieve the *κ* of the order of 0.8. The Polish clinical recommendations specifically mentioned only testing all individuals with severe infections [30]. In addition testing is provided to health care workers. The severe course corresponds to approximately 18% of all infections [18]. Therefore, the *κ* = 0.8 scenario is unlikely to be realistic in Poland. The plausible *κ* in our country during the summer 2020 lied close to 0.5. This is supported by comparing the model predictions to the observed data, which fit the *κ* = 0.5 scenario. Of note, for these scenarios the model shows that the control of the epidemic is largely achieved through suppression of *β*. In case of relaxation of social contact restrictions, the efforts should be focused on increasing the level of testing in order to decrease the proportion of undiagnosed cases as well as maintaining or increasing the effectiveness of quarantine. For smaller *κ*, even substantially increasing the effectiveness of quarantine does not allow to go back to the level of social contacts from before the epidemic (*β*_1_). However, during the summer 2020 the restrictions were lifted in Poland, without increasing the testing rates. Moreover, testing indications were further restricted in September, when also the schools were re-opened and teleworking was not longer required. This led to the scenario of rapid incidence increase and reaching the top contact tracing capacity. The maximum number of quarantined individuals reached 450 thousands in November (https://www.gov.pl/web/koronawirus/wykaz-zarazen-koronawirusem-sars-cov-2), corresponding to approximately 45 thousands of individuals put on quarantine each day, close to our assumption on the maximal quarantine capacity (50 thousands). This resulted in devastating epidemic wave in November-December 2020.

Our approach has several limitations. We do not consider the possibility of reduced transmission from undiagnosed cases who are more likely to be asymptomatic or paucisymtopmatic (*β*_*u*_ < *β*_*d*_). However, we lacked sufficient data to include this additional parameter. We calibrated our model only to diagnosed cases counts, which were officially available. Calibration to mortality data is another approach successfully implemented in e.g. [9]. As there were relatively fewer fatalities in Poland and little data on clinical progression we decided on simplified model without explicit modelling of the outcomes. Furthermore, we did not consider the sub-optimal adherence to quarantine. It is likely that some individuals would not fully comply to strict quarantine rules. However, only anecdotal evidence for such phenomenon was available. In our model it would decrease the effective *αθ*, which was chosen to fit to observed number of people put in quarantine. Another important factor of an outbreak of Covid-19 epidemic, especially at its early stage, which is not in the scope of our analysis, is the presence of so-called *super-spreaders*. The super-spreader phenomenon was taken into account for example in the work of Kochańczyk et al. [31], who showed that it leads to higher estimated values of R compared to the case when super-spreading is not taken into account.

In conclusion we have presented a simple model, which allows to understand the effects of testing, contact tracing and quarantining of the contacts. We apply the model to the data in Poland and we show that despite a substantial impact of contact tracing and quarantine, it is unlikely that the control of the epidemic could be achieved without any reduction of social contacts.

## Supporting information

S1 AppendixIt contains information on the optimization algorithm and initial data, rationale for the choice of fixed parameters and stability analysis of computation of R.(PDF)
