# Supplementary material for: On limits of contact tracing in epidemic control
Source: PLoS One. 2021 Aug 18;16(8):e0256180. doi: 10.1371/journal.pone.0256180 (PMC8372969; doi:10.1371/journal.pone.0256180)
Supplement: S1 Appendix — (PDF) [file pone.0256180.s001.pdf]

Supplementary information to:

On limits of contact tracing in epidemic control

by

Tomasz Piasecki, Piotr B. Mucha, Magdalena Rosińska

A Appendix

A.1 Optimization algorithm and initial data

In order to fit the values $\beta_1, \beta_2, \beta_3$ we use a standard gradient descent algorithm. Namely, we define error function as

$$E(\kappa) = \left[\frac{\sum_{t=1}^{54} [R_d(\kappa, t) - data(t)]^2}{\sum_{t=1}^{54} |data(t)|^2} \right]^{1/2}, \tag{A.1}$$

where $\{R_d(\kappa, t)\}_{t=1}^{54}$ is the vector of computed values of R_d and $\{data(t)\}_{t=1}^{54}$ the vector of data (cumulative number of confirmed cases).

At each step we approximate the gradient of the error function with respect to $\beta_1, \beta_2, \beta_3$ by differential quotients and move in the direction opposite to the gradient. The algorithm reveals a good performance provided we start sufficiently close to the minimum, which is not difficult to ensure in our case.

It remains to choose the initial data. A closer look on results of simulations shows that the choice of initial data mostly influence the fitting in the beginning of period under consideration and hence the value of β_1, while for analysis of future scenarios β_3 is the most important. Taking all this into account we do not struggle for sharp optimization of data fitting with respect to initial data and restrict to the following heuristic choice. It is natural to assume $I_u(0) = \frac{1-\kappa}{\kappa} I_d(0)$. Concerning the choice of $E(0)$ we assume it in a form $E(0) = m(I_d(0) + I_u(0))$. We set initial values $I_d(0) \in \{10, 20, 30\}$ and for each value we set $I_u(0)$ according to the above formula and three values of $E(0)$ corresponding to $m \in \{2, 3, 4\}$. For each of these 9 combinations we run the optimization algorithm looking for the best fit of $\beta_i, i = 1 \ldots 3$. We have repeated this approach for $\kappa \in \{0.2, 0.5, 0.8\}$. It turns in that for all values of κ the best fit was obtained for $I_d(0) = 20$ and $m = 2$. More careful analysis around $I_d(0) = 20$ did not improve the quality of fitting, therefore:

$$I_d(0) = 20, \quad I_u(0) = \frac{1-\kappa}{\kappa} I_d(0), \quad E(0) = 2(I_d(0) + I_u(0))$$

is our final choice. We obtain the following fitting error defined by (A.1):

κ	0.2	0.5	0.8
$E(\kappa)$	0.0077	0.0079	0.0084

Table A.1: Errors of data fit.

To close the set of initial data we put $K(s) = 0$ for $-T \leq s \leq 0$, which is a requirement of ODE with delay T.

A.2 Choice of fixed parameters

1. The parameter σ describes the rate of transition from non-infectious incubation state E into the infectious states I_d or I_u. The median incubation time from exposure till the onset of symptoms was estimated at 4 to 5 days [5, 6, 7]. However, there exists evidence that typically infectivity preceeds symptoms, by 1 to 3 days [8, 1, 2].

A modelling study identified the rate of transition between the non-infectious and infectious states at $\frac{1}{3.69}$ [10], which corresponds to an average time lag of 3.69 days untill the case becomes infectious.

2. The parameter γ_u represents the period of infectivity during the natural course of disease. We discuss the period of infectivity, especially as applied to mild cases. The median duration of viral shedding was estimated among 113 Chinese hospitalized patients. Overall it was 17 days, but it was shorter among cases with milder clinical course [14]. A study among 23 patients in Hong Kong confirmed viral shedding longer than 20 days among a third of patients, although the peak level of shedding was noted during the first week of infection [15]. In the mission report from China WHO reports viral shedding in mild and moderate cases to last 7 - 12 days from symptom onset. Among younger and asymptomatic or mild cases the shedding may be shorter: in a study among 24 initially asymptomatic youngsters the median duration was 9.5 days [9].

3. The value of κ generally depends on the testing policy. However, recommended testing policies often rely on the presence of respiratory symptoms. This is also the case in Poland. It was observed that some infected people never develop symptoms, although the precised rate of such truly asymptomatic infections is still under investigation. Some studies may be biased by a too short follow-up time. A small study among residents of a long-term care skilled nursing facility found that even though more than half of individuals with confirmed infection were asymptomatic at the time of test, majority of them subsequently developed symptoms. The proportion of people who remain asymptomatic may be higher among younger individuals [9]. A study among Japanese nationals repatriated from Wuhan suggests the proportion of asymptomatic infections is about 30% [11]. An analysis among the passengers of Diamond Princess ship, where a COVID-19 outbreak occurred, taking into account this delayed onset of symptoms estimated the proportion of asymptomatic infections to be about 18%, even though almost 50% were asymptomatic on initial test. In addition, large scale screening implemented in Italian village Vo'Euganeo indicated that 50% to 75% of infected individuals did not report symptoms [3]. Similarly, in population screening in Iceland 50% were asymptomatic at the time of screening [12]. It may be stipulated that some of the people diagnosed through screening developed symptoms latter, consistently with the findings from the Diamond Princess study.

On the other hand a sizable proportion of infected people, especially at younger ages, experience only mild symptoms, for which they may not seek medical attention. In the study of Li [10], the proportion of undocumented cases was estimated as 86%.

4. The parameter γ_d was estimated basing on a sample of case-based data available in routine surveillance, by fitting gamma distribution to the time from onset to diagnosis, for cases who were not in quarantine before diagnosis. Time from onset to diagnosis was estimated based on surveillance data available in the Epidemiological Reports Registration System for COVID-19, as of 28.04.2020.The system collects epidemiological data on cases diagnosed in Poland and is operated by local public health departments. All cases eventually are entered into the database. However, substantial reporting delays are noted. There were altogether 4976 cases registered in the system, including 1995 (40.1%), who did not have symptoms at the time of diagnosis. Plausible onset date and plausible diagnosis date were available for 2884 cases (96.7% of 2981 cases that were not asymptomatic)

Gamma distribution was fitted by maximum likelihood to cases who were not diagnosed in quarantine. The observed and fitted distributions are shown below (figure A.1).

We next fitted gamma-regression model with week of diagnosis as an explanatory variable. We found no significant trend in time. We therefore adopted the average time from onset to diagnosis to be 4.6 days, and taking into account the probability of asymptomatic spread we assumed the parameter γ_d to be 1/5.5.

5. Next we base θ on available data. We calculate prevalence of infection among the quarantined individuals, according to data published by the Chief Sanitary Inspectorate on the number of cases diagnosed among quarantined people and the total number of quarantined. We used a series of data 8.04 – 20.04 to estimate a likely value of θ. We chose this time period due to data availability. Data are shown on the figure below. During this time period there was an increasing trend in the proportion of diagnosed from 0.5% to 0.8% A.2. We presume that this parameter could change with changing procedures of contact tracing and testing. However, since no detailed data were available, for the modelling purposes we chose a simplifying assumption that θ is stable (i.e. we always take a similar group of contacts under quarantine) selecting an average value of 0.6%.

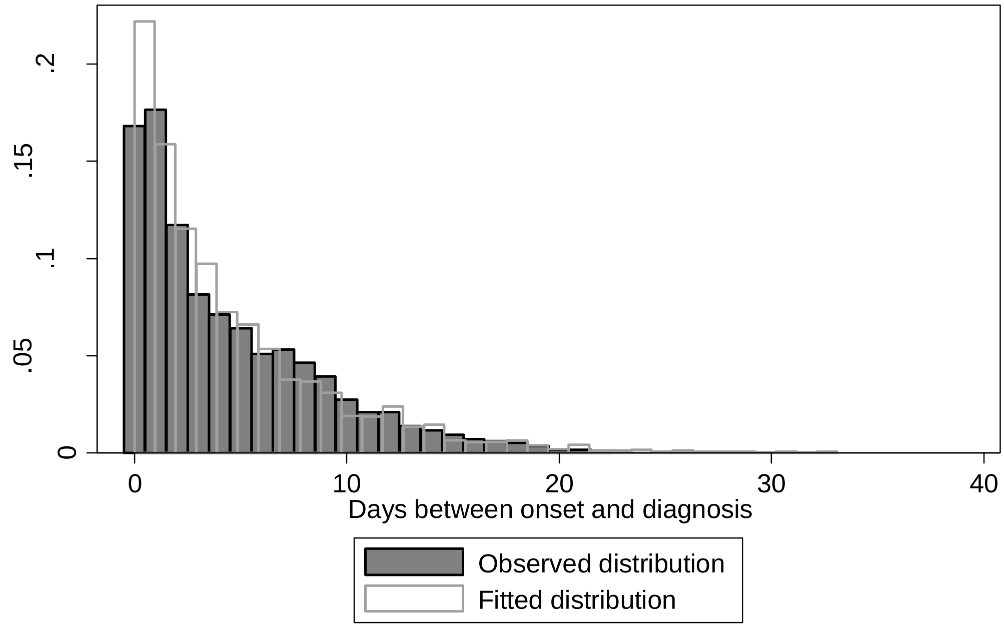

Figure A.1: Observed and fitter distribution of time between onset and diagnosis among symptomatic cases, who where diagnosed outside of quarantine. The fitted gamma distribution has the following parameters: shape = 0.91, scale = 5.06

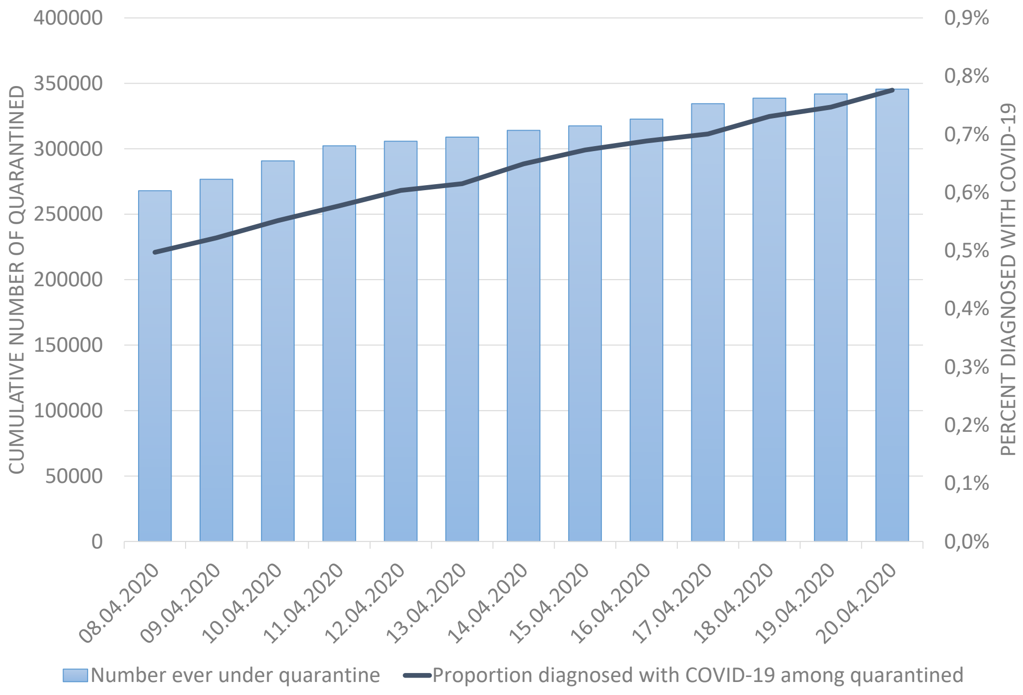

Figure A.2: Data on the population undergoing quarantine and the percent diagnosed with COVID-19 in this population

This proportion could be also viewed as attack rate among the contacts of cases. The proportion in Poland is in line with what was observed in Korea, where an estimated attack rate was 0.55% overall and 7.56% among household contacts [13], although household attack rate was higher (>

19%) in other studies [16].

6. Furthermore we fix the parameter α. Here we make another simplification assuming this parameter to be constant. The main difficulty is a lack of precise data concerning the number of newly quarantined people per day, distinguishing between reasons of quarantine (travel related or contact tracing relate). At the beginning of epidemic in Poland the average amount of quarantined following one diagnosed case was definitely higher. Moreover, people coming back from abroad were subject to obligatory quarantine starting from March 16 and constituted a considerable part of quarantined in the second half of March and beginning of April. In particular, around 54 000 Polish citizens staying abroad came back within a special program of charter flights operated by Polish Airlines which ended on April 5. We can assume that after this date the ratio of people coming from abroad among all people subject to quarantine was negligible. As our model does not take migration into account, we have to take into account only quarantine from contact. For above reasons, for fitting α we restrict our analysis only to a period of 2 weeks of April. Assuming already $\theta = 0.006$ we then choose α minimizing the square error between the number of quarantined from the data and computed K(t). This way we obtain $\alpha = 75$.

A.3 Confidence intervals

Bootstrap. To estimate confidence intervals we use a method of parametric bootstrap. We generate $M = 200$ sequences of perturbed data assuming that for each time $t \in \{1, 54\}$ the increment of R (i.e. daily number of new diagnoses) is a random number from Poisson distribution with mean value equal to increment of observed data.

Model parameters are also perturbed, see below. For each series of perturbed data we estimate the values of β_i and take estimated confidence intervals as appropriate quantiles of obtained sets.

In order to estimate confidence intervals for $R_d(t)$, we proceed as follows. For each sequence of perturbed data we compute fitted $R_d(t)$. This way we obtain a set of curves

$$\{R_d^{(k)}(t)\}_{k=1,\dots,200}^{t=1,\dots,54}.$$

Then for each time instant $t \in \{1, 54\}$ we set lower and upper bounds of the confidence interval of $R_d(t)$ as appropriate quantiles of the set $\{R_d^{(k)}(t)\}_{k=1}^{200}$. Analogously we compute the confidence intervals for $R_u(t)$.

Distribution of parameters. Following other Authors [17] as well as experimental data, for the uncertainty analysis we used the following distributions of the parameters.

1. $1/\gamma_d \sim Gamma(a_1, b_1)$, where the shape parameter, $a_1 = 1.05$ and scale parameter $b_1 = 5.23$
2. $1/\gamma_u \sim Gamma(a_2, b_2)$, where the shape parameter, $a_2 = 2$ and scale parameter $b_2 = 5$
3. $1/\sigma \sim Gamma(a_3, b_3)$, where the shape parameter, $a_2 = 2$ and scale parameter $b_2 = 1.75$
4. $\alpha \sim Poisson(\alpha_0)$, where we assume constant $\alpha_0 = 75$;
5. θ - is not sampled for the uncertainty analysis. As the results depend on the quantity $\alpha\theta$, we rely on the distribution of α.

We take $N = 200$ (approximate average of daily number of diagnosed cases from the data). We approximate the mean value of N samples from Gamma distribution using Central Limit Theorem. Namely, we generate

$$\frac{1}{\gamma_d} \sim \mathcal{N}(a_1 b_1, \frac{a_1 b_1^2}{N}), \quad \frac{1}{\gamma_u} \sim \mathcal{N}(a_2 b_2, \frac{a_2 b_2^2}{N}), \quad \frac{1}{\sigma} \sim \mathcal{N}(a_3 b_3, \frac{a_3 b_3^2}{N}).$$

A.4 Stability analysis - computation of $\mathcal{R}$

Based on the classical approach to epidemiological models we address the basic question concerning the propagation of the disease. Namely, how many persons are infected by one infectious individual, a quantity which is usually called reproductive number, $\mathcal{R}$. In order to compute this quantity we

use the approach from [4]. Recall, the system reads

$$
\begin{aligned}
&\dot S(t) = -\tfrac{S(t)}{N}(\beta_d I_d(t) + \beta_u I_u(t)) - (1-\theta)K(t) + (1-\theta)K(t-T),\\
&\dot E(t) = \tfrac{S(t)}{N}(\beta_d I_d(t) + \beta_u I_u(t)) - \sigma E(t) - \theta K(t)\\
&\dot I_d(t) = \kappa\sigma E(t) - \gamma_d I_d(t),\\
&\dot I_u(t) = (1-\kappa)\sigma E(t) - \gamma_u I_u(t),\\
&\dot R_d(t) = \gamma_d I_d(t) + \theta K(t-T),\\
&\dot R_u(t) = \gamma_u I_u(t),\\
&\dot Q(t) = K(t) - K(t-T),\\
&\text{where} \quad K(t) = \min\{\tfrac{S(t)}{S(t)+R_u(t)}\alpha\gamma_d I_d(t), K_{max}\}, \quad \text{and} \quad \alpha, \beta_d, \beta_u, \gamma_d, \gamma_u, \theta, T \geq 0.
\end{aligned}
\tag{A.2}
$$

We look then at the system assuming $S \sim N$ and E, I_d, I_u are close to zero, then we consider the following linearization

$$
\begin{aligned}
\dot E(t) &= \beta_d I_d(t) + \beta_u I_u(t) - \sigma E(t) - \theta\alpha\gamma_d I_d(t),\\
\dot I_d(t) &= \kappa\sigma E(t) - \gamma_d I_d(t),\\
\dot I_u(t) &= (1-\kappa)\sigma E(t) - \gamma_u I_u(t).
\end{aligned}
\tag{A.3}
$$

Note that in the above subsystem there are no delay effects. We write (A.3) as

$$\dot x = (T - \Sigma)x \tag{A.4}$$

where $x = (E, I_d, I_u)^T$, T is the transmission matrix and Σ – the transition matrix. T has nonnegative entries and Σ is lower triangular with strictly positive eigenvalues:

$$
T = \begin{pmatrix} 0 & \beta_d - \theta\alpha\gamma_d & \beta_u \\ 0 & 0 & 0 \\ 0 & 0 & 0 \end{pmatrix}, \qquad \Sigma = \begin{pmatrix} \sigma & 0 & 0 \\ -\kappa\sigma & \gamma_d & 0 \\ -(1-\kappa)\sigma & 0 & \gamma_u. \end{pmatrix}
\tag{A.5}
$$

The system (A.4) can be rewritten as

$$\dot x = -(Id - T\Sigma^{-1})\Sigma x. \tag{A.6}$$

Then one deduces (see [4]) that if we define $\mathcal{R} = \max\{\text{eigenvalues of } \; T\Sigma^{-1}\}$ then

the system is stable for $\mathcal{R} < 1$ and it is unstable for $\mathcal{R} > 1$.

Stability of system (A.6) means that the whole vector (E, I_d, I_u) is going to zero, it follows that the main system (A.2) also tends to the zero solution for (E, I_d, I_u). Instability implies that for "almost all" small data, the vector (E, I_d, I_u) is growing in time (exponentially fast), causing the nonlinear system also evolves far away from the trivial state, i.e. E, I_d, I_u rapidly grow.

By (A.5) we have

$$
T\Sigma^{-1} = \begin{pmatrix} \frac{\kappa(\beta_d - \theta\alpha\gamma_d)}{\gamma_d} + \frac{(1-\kappa)\beta_u}{\gamma_u} & \frac{\beta_d - \theta\alpha\gamma_d}{\gamma_d} & \frac{\beta_u}{\gamma_u} \\ 0 & 0 & 0 \\ 0 & 0 & 0 \end{pmatrix}.
$$

Hence the stability of our system is determined by the following factor:

$$\mathcal{R} = \frac{\kappa\beta_d}{\gamma_d} + \frac{(1-\kappa)\beta_u}{\gamma_u} - \kappa\theta\alpha. \tag{A.7}$$

To make a final comment, let us note that in case of spread of pandemia, as R_d, R_u grow, the above analysis become less reliable. Recall that β is normalized by N, so as S/N is not close to one and the analysis of stability becomes more complex.

Acknowledgments. This work was partially supported by the Polish National Science Centre's grant No2018/30/M/ST1/00340 (HARMONIA).

References

[1] Tong ZD, Tang A, Li KF, Li P, Wang HL, Yi JP, Zhang YL, Yan JB. Potential Presymptomatic Transmission of SARS-CoV-2, Zhejiang Province, China, 2020. Emerg. Infect. Dis. 2020;26:1052-1054. https://doi.org/10.3201/eid2605.200198.

[2] Huang L, Zhang X, Zhang X, Wei Z, Zhang L, Xu J, Liang P, Xu PY, Zhang C, Xu PA. Rapid asymptomatic transmission of COVID-19 during the incubation period demonstrating strong infectivity in a cluster of youngsters aged 16-23 years outside Wuhan and characteristics of young patients with COVID-19: a prospective contact-tracing study. J Infect. 2020; pii: S0163-4453(20)30117-1. https://doi.org/10.1016/j.jinf.2020.03.006.

[3] Day M. Covid-19: identifying and isolating asymptomatic people helped eliminate virus in Italian village. BMJ 2020; 368 :m1165.

[4] Diekmann O, Heesterbeek JAP, Roberts MG. The construction of next-generation matrices for compartmental epidemic models. J. R. Soc. Interface 2010; 7:873–885. https://doi.org/10.1098/rsif.2009.0386.

[5] Guan WJ, Ni ZY, Hu Y, et al. Clinical Characteristics of Coronavirus Disease 2019 in China. N Engl J Med. 2020;382:1708-1720. https://doi.org/10.1056/NEJMoa200203.

[6] Li Q, Guan X, Wu P, et al. Early Transmission Dynamics in Wuhan, China, of Novel Coronavirus-Infected Pneumonia. N Engl J Med. 2020;382:1199-1207. https://doi.org/10.1056/NEJMoa2001316.

[7] Lauer SA, Grantz KH, Bi Q, et al. The Incubation Period of Coronavirus Disease 2019 (COVID-19) From Publicly Reported Confirmed Cases: Estimation and Application. Ann Intern Med. 2020;172:577-582.https://doi.org/10.7326/M20-050.

[8] Wei WE, Li Z, Chiew CJ, Yong SE, Toh MP, Lee VJ. Presymptomatic Transmission of SARS-CoV-2 - Singapore, January 23-March 16, 2020. MMWR Morb Mortal Wkly Rep. 2020;69:411-415. https://doi.org/10.15585/mmwr.mm6914e1.

[9] Hu Z, Song C, Xu C, Jin G, Chen Y, Xu X, Ma H, Chen W, Lin Y, Zheng Y, Wang J,Hu Z, Yi Y, Shen H. Clinical characteristics of 24 asymptomatic infections with COVID-19 screened among close contacts in Nanjing, China. Sci China Life Sci. 2020; https://doi.org/10.1007/s11427-020-1661-4.

[10] Li R, Pei S, Chen B, Song Y, Zhang T, Yang W, Shaman J. Substantial undocumented infection facilitates the rapid dissemination of novel coronavirus (SARS-CoV2). Science. 2020; pii: eabb3221. htpps://doi.org/10.1126/science.abb3221.

[11] Nishiura H, Kobayashi T, Suzuki A, Jung SM, Hayashi K, Kinoshita R, Yang Y,Yuan B, Akhmetzhanov AR, Linton NM, Miyama T. Estimation of the asymptomatic ratio of novel coronavirus infections (COVID-19). Int J Infect Dis; pii: S1201-9712(20)30139-9. https://doi.org/10.1016/j.ijid.2020.03.020.

[12] John T. Iceland lab's testing suggests 50% of coronavirus cases have no symptoms. CNN. 2020. https://edition.cnn.com/2020/04/01/europe/iceland-testing-coronavirus-intl/index.html. Accessed 15 May 2020.

[13] COVID-19 National Emergency Response Center, Epidemiology and Case Management Team, Korea Centers for Disease Control and Prevention. Coronavirus Disease-19: Summary of 2,370 Contact Investigations of the First 30 Cases in the Republic of Korea. Osong Public Health Res Perspect. 2020;11(2):81-84.

[14] Xu K, Chen Y, Yuan J, Yi P, Ding C, Wu W, Li Y, Ni Q, Zou R, Li X, Xu M, Zhang Y, Zhao H, Zhang X, Yu L, Su J, Lang G, Liu J, Wu X, Guo Y, Tao J, Shi D, Yu L, Cao Q, Ruan B, Liu L, Wang Z, Xu Y, Liu Y, Sheng J, Li L. Factors associated with prolonged viral RNA shedding in patients with COVID-19. Clin Infect Dis. 2020; pii: ciaa351. https://doi.org/10.1093/cid/ciaa351.

[15] To KK, Tsang OT, Leung WS, Tam AR, Wu TC, Lung DC, Yip CC, Cai JP, Chan JM, Chik TS, Lau DP, Choi CY, Chen LL, Chan WM, Chan KH, Ip JD, Ng AC, Poon RW, Luo CT, Cheng VC, Chan JF, Hung IF, Chen Z, Chen H, Yuen KY. Temporal profiles of viral load in posterior oropharyngeal saliva samples and serum antibody responses during infection by SARS-CoV-2: an observational cohort study. Lancet Infect Dis. 2020; pii: S1473-3099(20)30196-1. https://doi.org/10.1016/S1473-3099(20)30196-1.

[16] Qin-Long Jing, Ming-Jin Liu, Jun Yuan, Zhou-Bin Zhang, An-Ran Zhang, Natalie E Dean, Lei Luo, Meng-Meng Ma, Ira Longini, Eben Kenah, Ying Lu, Yu Ma, Neda Jalali, Li-Qun Fang, Zhi-Cong Yang, Yang Yang. Household Secondary Attack Rate of COVID-19 and Associated Determinants. medRxiv. 2020; https://doi.org/10.1101/2020.04.11.20056010.

[17] Kucharski AJ, Russell TW, Diamond C, Liu Y, Edmunds J, Funk S, Eggo RM; Centre for Mathematical Modelling of Infectious Diseases COVID-19 working group. Early dynamics of transmission and control of COVID-19: a mathematical modelling study. Lancet Infect Dis. 2020;20:553-558. https://doi.org/10.1016/S1473-3099(20)30144-4.
